# Role of psychosocial work factors in the relation between becoming a caregiver and changes in health behaviour: results from the Whitehall II cohort study

**DOI:** 10.1136/jech-2015-206463

**Published:** 2016-05-23

**Authors:** Nadya Dich, Jenny Head, Naja Hulvej Rod

**Affiliations:** 1 Department of Public Health, University of Copenhagen, Copenhagen, Denmark; 2 Department of Epidemiology and Public Health, University College London, London, UK; 3 Department of Public Health, University of Copenhagen, Copenhagen, Denmark

**Keywords:** Work stress, HEALTH BEHAVIOUR, STRESS

## Abstract

**Background:**

The present study tested the effects of becoming a caregiver combined with adverse working conditions on changes in health behaviours.

**Methods:**

Participants were 5419 British civil servants from the Whitehall II cohort study who were not caregivers at baseline (phase 3, 1991–1994). Psychosocial work factors were assessed at baseline. Phase 4 questionnaire (1995–1996) was used to identify participants who became caregivers to an aged or disabled relative. Smoking, alcohol consumption and exercise were assessed at baseline and follow-up (phase 5, 1997–1999).

**Results:**

Those who became caregivers were more likely to increase frequency of alcohol consumption, but only if they also reported low decision latitude at work (OR= 1.65, 95% CI 1.15 to 2.37 compared with non-caregivers with average decision latitude), or belonged to low occupational social class (OR=2.38, 95% CI 1.17 to 4.78 compared with non-caregivers of high occupational social class). Caregivers were more likely to quit smoking if job demands were low (OR=2.92; 95% CI 1.07 to 7.92 compared with non-caregivers with low job demands), or if social support at work was high (OR=2.99, 95% CI 1.01 to 8.86 compared with caregivers with average social support). There was no effect of caregiving on reducing exercise below recommended number of hours per week, or on drinking above recommended number of units per week, regardless of working conditions.

**Conclusions:**

The findings underscore the importance of a well-balanced work environment as a resource for people exposed to increased family demands.

Health-related behaviours such as smoking, alcohol consumption and inactivity contribute to the aetiology of many chronic diseases including cardiovascular disease, diabetes and cancer.^1^ As health behaviours are one of the main targets of public health interventions,^2^ understanding the causes of positive and negative changes in health behaviours is an important public health issue.

Psychosocial stress as a potential cause of changes in health behaviour has been the focus of researchers' attention for a long time.^3^ Indeed, stress increases vulnerability to addiction, making individuals more likely to develop alcohol, nicotine or other substance dependency, and less likely to quit existing addictive behaviours.^4–6^ Moreover, stress depletes self-regulation and motivation^7^ and may thereby increase the risk of an unhealthy behaviour, for example, poor diet and low physical inactivity.^6^ ^8^ ^9^

Unfavourable psychosocial workplace conditions are among most researched stressors, with a number of studies finding a link between work stress and adverse health behaviours.^10–13^ Recent meta-analyses combining data from multiple European cohort studies show modest size associations between job strain (a combination of high demands and low control at work) and higher likelihood of being or becoming inactive,^10^ and lower likelihood of adopting a healthy lifestyle.^12^ Cross-sectional association between job strain and tobacco and alcohol consumption have also been found.^11^ ^13^

Less is known about the role of increased family stress in health behaviour changes, in particular, the role of informal caregiving. With the number of informal caregivers rising as a result of population ageing, the potential adverse effects of caregiving become an important public health issue. Previous literature has emphasised both positive and negative effects of caregiving on health and well-being.^14^ ^15^ On the one hand, providing care to a relative might feel meaningful, fulfilling and rewarding, with positive aspects of caregiving associated with better mental health.^15^ On the other hand, the emotional strain of caregiving may also adversely affect caregivers' well-being and health.^14^ ^16^ However, the effects of becoming a caregiver on changes in health behaviours have not been extensively studied, and results have been mixed. For instance, a recent review of literature on health behaviours and caring for patients with cancer revealed both detrimental and positive changes in caregivers' health behaviours.^17^

The conflicting findings regarding caregiving and health behaviours may be partly due to the fact that consequences of becoming a caregiver depend on the caregiver's more general life circumstances, including those not related to family. Indeed, it has been suggested that additional exposure to other stressors, such as a demanding job, may exacerbate the burden of family obligations on health;^18^ however, to the best of our knowledge, the combined effects of becoming a caregiver and work stress on changes in health behaviours have not previously been investigated. The present study tested the hypothesis that the effects of becoming a caregiver may depend on the psychosocial working conditions prior to the increased family demands.

This hypothesis is also in line with the emerging evidence that work and family factors might interact in predicting physical and mental health. For instance, Madsen *et al*^19^ recently showed that low social support in private life and job strain were associated with higher risk of developing severe depressive symptoms, but only in combination and not by themselves. Melchior *et al*^20^ found that people with the highest number of demands at work and at home had the highest rates of sickness absence with psychiatric diseases. A recent study based on Whitehall II also showed that detrimental effects of high caregiving burden on allostatic load, a measure of cumulative physiological dysregulation, appeared to be more pronounced in individuals who reported job strain compared to those who did not report job strain.^21^ With respect to interactions of work and family factors in health behaviour changes, one study showed that among women, a combination of full-time employment and marital conflict was associated with increased alcohol use.^22^

The present study used longitudinal data with repeated measures on caregiving and lifestyle factors to investigate the combined effects of becoming a caregiver to an old or disabled relative and psychosocial work factors prior to becoming a caregiver on changes in alcohol consumption, smoking and physical activity.

## Method

### Study design and participants

The study was based on data from the Whitehall II cohort study. The original sample (phase 1, recruited in 1985–1988) included 10 308 British civil service workers aged 35–55 years.^23^ Follow-up questionnaires were administered at every subsequent phase approximately every 2 years. Phase 3 (1991–1994) served as the baseline in the present study, as information about caregiving was not available for earlier phases. Data from the following phase (phase 4, 1995–1996) were used to identify subjects who became caregivers after the baseline assessment. Questionnaire data from phases 3 and 5 (1997–1999) were used to assess health behaviours before and after becoming a caregiver.

In total 7054 returned questionnaires at phases 3 and 5 and had information about caregiving at phase 4. To investigate changes in health behaviours related to becoming a caregiver, 764 participants who already were caregivers at phase 3 were excluded. An additional 871 subjects were excluded due to missing information on psychosocial work factors or covariates. The final sample consisted of 5419 subjects (29% women, average age=49 years, ranging from 39 to 62).

The Whitehall II study is approved by the Joint University College London and University College London Hospital Committees on the Ethics of Human Research. All participants were asked to give written informed consent.

### Caregiving

Since none of the included participants were caregivers at baseline, reporting taking care of an aged or disabled relative at phase 4 was used as the indicator for becoming a caregiver.

### Work characteristics

Work characteristics were assessed at baseline. The Job Content Instrument,^24^ a validated measure of psychosocial workplace characteristics, was used in the present study. The instrument comprises three scales: psychological job demands, such as working under time pressure (Cronbach's α=0.67, 4 items), decision latitude, reflecting the ability to influence work-related decisions and use one's own skills (α=0.84, 15 items), and support at work, reflecting help and support from colleagues and supervisors (α=0.79, 6 items). Additionally, occupational social class (administrators, professionals and executives, clerical and office support) was used as a global indicator of psychosocial conditions at work.^25^

### Changes in health behaviours

#### Changes in alcohol consumption

At baseline and follow-up, participants reported frequency of alcohol consumption in the past year (more than twice a day, daily, once a week, once a month, on special occasions, not at all), and the number of alcoholic drinks consumed in the past week. We chose to use the former as an indicator of changes in alcohol consumption, as we reasoned that a measure across the whole year would reflect more stable drinking habits than a weekly measure, which may be subject to considerable fluctuations. A binary variable was constructed reflecting increases in frequency of alcohol consumption between baseline and follow-up (yes/no). On the other hand, reports of drinking during the past year may be less accurate than reports of drinking in the past week due to fading memory. Furthermore, relative increases in frequency do not say anything about the amount of drinking and, therefore, are more difficult to interpret in terms of clinical relevance. Therefore, in addition, we also constructed a measure reflecting increased drinking to levels above recommended maximum (≥21 alcoholic drinks for men and ≥14 alcoholic drinks for women per week), based on the number of drinks consumed in the past week.

#### Smoking cessation

At baseline and follow-up, participants reported whether or not they smoked, based on which a binary variable reflecting quitting smoking was constructed.

#### Changes in physical activity

At phase 3 (baseline), mild, moderate and vigorous physical activity had been previously assessed based on questions about the average number of hours a week participants engaged in ‘mildly energetic’ (eg, weeding, general housework, bicycle repair), ‘moderately energetic’ (eg, dancing, cycling, leisurely swimming), and ‘vigorous’ (eg, running, hard swimming, playing squash) physical activity. At phase 5, hours of physical activity per week had been computed based on 20 questions about frequency and duration of walking, cycling, sports, gardening, housework and home maintenance. Each of the 20 physical activities had been assigned a metabolic equivalent (MET).^26^ MET values ranging from 3 to 6 (eg, cycling, gardening) were used as equivalent of moderate physical activity. MET values of 6 or above (eg, sports) were used as indicators of vigorous physical activity. Basing on this information, we calculated for each of the phases, whether or not the participants met the recommended minimum amount of exercise according to WHO guidelines (at least 150 min a week of moderate exercise, or at least 75 min of vigorous exercise, or an equivalent combination of the two^27^). A binary variable was then created reflecting whether participants reduced the amount of exercise below the recommended minimum within the follow-up period.

### Statistical analyses

Logistic regression was used to model increased alcohol consumption, smoking cessation, and reduced amount of exercise. A main-effect model was fitted to data first, where becoming a caregiver, job demands, decision latitude, social support at work and social class were entered simultaneously as predictors. To assess the combined effects of becoming a caregiver and occupational factors, several interaction models were fitted. In each of these models, the product of becoming a caregiver and one of the occupational factors was added to main effect model to test for multiplicative interaction. The results are also presented as a joint effect with a common reference category, as recommended in STROBE (Strengthening the Reporting of Observational Studies in Epidemiology) guidelines.^28^

In all our models, we included age, gender and marital status as potential confounders, as those factors can influence perception of working conditions, taking on caregiving roles as well as health behaviours. Furthermore, perception of working conditions, health behaviours, and the ability to provide care to a relative may all depend on physical and mental health. On the other hand, health problems may also be consequences of working conditions, and thus, adjusting for them would constitute overadjustment. Therefore, we controlled for baseline health—depressive symptoms, measured using a depression subscale of General Health Questionnaire,^29^ and self-reported long-standing illness at baseline (yes/no)—in a separate set of models.

## Results

Six per cent of the participants (N=304) became caregivers over a 2-year period. Table 1 presents the distribution of study variables among those who became caregivers and those who did not. Among those who became caregivers, a slightly higher proportion reported high job demands, low decision latitude and low social support. Becoming a caregiver was not related to socioeconomic class.

**Table 1:** Distribution of study variables

	Sample size (total/those who became caregivers)		Among those who did not become caregivers (%)	Among those who became caregivers (%)
Social class	5419/304	Administrative	2090 (41)	123 (40)
		Professional/executive	2302 (45)	142 (47)
		Clerical/support	723 (14)	39 (13)
High job demands*	5419/304		2519 (49)	171 (56)
Low decision latitude*	5419/304		2595 (51)	164 (54)
Low social support*	5419/304		2220 (43)	150 (49)
Increased frequency of alcohol consumption	5031†/282		1165 (25)	71 (25)
Started to drink above recommended limits	4121‡/237		577 (15)	32 (14)
Quit smoking	626§/35		160 (27)	11 (31)
Reduced exercise below recommended amount	2697¶/140		805 (31)	48 (34)

*Based on sample median as a cut-off.

†388 participants were missing information on frequency of alcohol consumption.

‡Drank within recommended limits at baseline.

§Smoked at baseline.

¶Exercised the recommended amount at baseline.

Information on alcohol consumption was available for 5031 participants. Twenty-five per cent of these 5031 increased the frequency of alcohol consumption during follow-up. Eighty-two per cent of participants reported drinking below recommended maximum in the past week at baseline, of whom 15% reported drinking above recommended limits at follow-up. Information about smoking was available for 5176 participants. Thirteen per cent of the participants smoked at baseline, and 27% of those quit smoking by follow-up. Fifty-three per cent of all participants met the recommendation regarding the amount of exercise at baseline. Of those, 32% reduced exercise below the recommended amount by follow-up.

### Job factors, caregiving and changes in alcohol consumption

Table 2 shows the effects of baseline work factors and becoming a caregiver on alcohol consumption. Increased frequency of alcohol consumption was not related to any of these factors when they were modelled as main effects mutually adjusted for each other. However, the interaction analyses showed that the effect of becoming a caregiver depended on baseline decision latitude and occupational social class (table 3). While the level of decision latitude was not associated with alcohol intake among non-caregivers (OR=0.99; 95% CI 0.91 to 1.08 for low vs average decision latitude), those with low decision latitude who became caregivers were more likely to increase drinking compared with non-caregivers with average decision latitude (OR=1.65, 95% CI 1.15 to 2.37). At the same time, those who became caregivers were less likely to increase drinking if they also reported high decision latitude (OR=0.56, 95% CI 0.36 to 0.88).

**Table 2:** Effects of caregiving and psychosocial work factors on changes in health behaviours, adjusted for age, gender and marital status

	Increased frequency of alcohol consumption		Started to drink above recommended limit		Quit smoking		Reduced exercise below recommended amount	
	Nr. (%)	OR (95% CI)	Nr. (%)	OR (95% CI)	Nr. (%)	OR (95% CI)	Nr. (%)	OR (95% CI)
Became caregiver								
No	1165 (25)	(ref)	577 (15)	(ref)	160 (27)	(ref)	805 (31)	(ref)
Yes	71 (25)	1.03 (0.78 to 1.37)	32 (14)	0.91 (0.61 to 1.33)	11 (31)	1.26 (0.59 to 2.67)	48 (34)	1.03 (0.70 to 1.50)
Social class								
Administrative	517 (25)	(ref)	311 (19)	(ref)	61 (36)	(ref)	264 (29)	(ref)
Professional/executive	551 (24)	0.98 (0.84 to 1.15)	257 (14)	0.76 (0.62 to 0.94)	87 (27)	0.71 (0.46 to 1.11)	436 (35)	1.43 (1.17 to 1.76)
Clerical/support	168 (26)	0.99 (0.75 to 1.30)	41 (7)	0.42 (0.27 to 0.63)	23 (17)	0.41 (0.20 to 0.85)	153 (61)	2.57 (1.79 to 3.72)
Job demands*								
Low		1.01 (0.94 to 1.09)		0.92 (0.83 to 1.02)		0.81 (0.66 to 1.00)		1.00 (0.91 to 1.11)
Average		(ref)		(ref)		(ref)		(ref)
High		0.99 (0.92 to 1.06)		1.09 (0.98 to 1.20)		1.23 (1.00 to 1.51)		1.00 (0.90 to 1.10)
Decision latitude*								
Low		1.02 (0.94 to 1.11)		0.97 (0.86 to 1.08)		1.05 (0.84 to 1.32)		1.24 (1.12 to 1.39)
Average		(ref)		(ref)		(ref)		(ref)
High		0.98 (0.90 to 1.06)		1.04 (0.92 to 1.16)		0.95 (0.76 to 1.20)		0.80 (0.72 to 0.90)
Social support*								
Low		1.03 (0.96 to 1.10)		1.00 (0.91 to 1.10)		0.96 (0.80 to 1.16)		0.98 (0.89 to 1.07)
Average		(ref)		(ref)		(ref)		(ref)
High		0.97 (0.91 to 1.04)		1.00 (0.91 to 1.09)		1.04 (0.87 to 1.25)		1.02 (0.93 to 1.12)
Total sample/Became caregivers	5031/282		4121/237		626/35		2697/140	

*Continuous scale. Low and high values defined as 1 SD below and above the mean, respectively.

**Table 3:** Combined effects of caregiving and psychosocial work factors on changes in health behaviours, adjusted for age, gender and marital status

	OR (95% CI) increased frequency of alcohol consumption		OR (95% CI) started to drink above recommended limit	
	Became caregiver		Became caregiver	
	No	Yes	No	Yes
Job demands				
Low*	1.01 (0.94 to 1.08)	1.18 (0.81 to 1.73)	0.91 (0.82 to 1.01)	1.04 (0.59 to 1.81)
Average	(ref)	1.04 (0.79 to 1.37)	(ref)	0.94 (0.64 to 1.39)
High*	0.99 (0.92 to 1.07)	0.91 (0.63 to 1.33)	1.10 (0.99 to 1.22)	0.85 (0.51 to 1.43)
p Value interaction	0.37		0.32	
Decision latitude				
Low*	0.99 (0.91 to 1.08)	1.65 (1.15 to 2.37)	0.97 (0.86 to 1.09)	0.86 (0.46 to 1.58)
Average	(ref)	0.96 (0.72 to 1.29)	(ref)	0.91 (0.62 to 1.35)
High*	1.01 (0.92 to 1.09)	0.56 (0.36 to 0.88)	1.03 (0.92 to 1.16)	0.97 (0.57 to 1.66)
p Value interaction	<0.001		0.89	
Social support				
Low*	1.01 (0.95 to 1.09)	1.26 (0.89 to 1.79)	1.00 (0.91 to 1.10)	0.96 (0.58 to 1.58)
Average	(ref)	0.99 (0.74 to 1.32)	(ref)	0.91 (0.61 to 1.34)
High*	0.99 (0.92 to 1.06)	0.78 (0.50 to 1.20)	1.00 (0.91 to 1.10)	0.86 (0.48 to 1.56)
p Value interaction	0.10		0.80	
Social class				
Administrative	(ref)	0.67 (0.40 to 1.07)	(ref)	0.94 (0.52 to 1.59)
Professional/executive	0.96 (0.82 to 1.12)	1.03 (0.68 to 1.54)	0.76 (0.62 to 0.94)	0.74 (0.41 to 1.28)
Clerical/support	0.91 (0.69 to 1.20)	2.38 (1.17 to 4.78)	0.43 (0.28 to 0.66)	0.18 (0.01 to 0.85)
p Value interaction	0.007		0.72	
Total sample	4749	282	3884	237
	**OR (95% CI) Quitting smoking**		**OR (95% CI) Reduced exercise below recommended amount**	
	**Became caregiver**		**Became caregiver**	
	**No**	**Yes**	**No**	**Yes**
Job demands				
Low*	0.77 (0.62 to 0.95)	2.24 (0.83 to 6.06)	1.00 (0.91 to 1.11)	1.07 (0.55 to 2.08)
Average	(ref)	1.20 (0.55 to 2.62)	(ref)	1.04 (0.70 to 1.54)
High*	1.30 (1.05 to 1.61)	0.64 (0.20 to 2.05)	1.00 (0.90 to 1.10)	1.00 (0.60 to 1.67)
p Value interaction	0.022		0.88	
Decision latitude				
Low*	1.04 (0.82 to 1.31)	1.53 (0.61 to 3.85)	1.25 (1.12 to 1.40)	1.13 (0.66 to 1.93)
Average	(ref)	1.19 (0.55 to 2.60)	(ref)	1.03 (0.71 to 1.51)
High*	0.97 (0.76 to 1.22)	0.93 (0.29 to 3.00)	0.80 (0.71 to 0.89)	0.94 (0.55 to 1.63)
p Value interaction	0.56		0.50	
Social support				
Low*	1.01 (0.83 to 1.22)	0.61 (0.19 to 1.95)	0.98 (0.89 to 1.08)	0.98 (0.59 to 1.61)
Average	(ref)	1.35 (0.63 to 2.98)	(ref)	1.03 (0.70 to 1.52)
High*	0.99 (0.82 to 1.20)	2.99 (1.01 to 8.86)	1.02 (0.93 to 1.12)	1.10 (0.61 to 1.95)
p Value interaction	0.054		0.85	
Social class				
Administrative	(ref)	0.54 (0.08 to 2.45)	(ref)	1.15 (0.59 to 2.10)
Professional/executive	0.67 (0.42 to 1.04)	1.49 (0.53 to 4.10)	1.44 (1.17 to 1.77)	1.52 (0.88 to 2.57)
Clerical/support	0.42 (0.20 to 0.88)	0.24 (0.01 to 1.44)	2.67 (1.83 to 3.89)	1.74 (0.56 to 5.33)
p Value interaction	0.24		0.68	
Total sample	591	35	2557	140

*Low and high defined as 1 SD below and above the mean, respectively.

Social class was not associated with increased alcohol consumption among non-caregivers (table 3). However, those who became caregivers and belonged to the low social class were more likely to increase drinking compared with non-caregivers in the highest social class (OR=2.38, 95% CI 1.17 to 4.78). A combination of becoming a caregiver with high job demands and low social support were not related to higher risk of increased drinking (table 3).

The pattern of results was different for drinking above the recommended limits. Only social class predicted drinking above limits, with highest social class being at highest risk (compared with the administrative (highest) social class, OR=0.67; 95% CI 0.42 to 1.04 for the professional/executive social class and OR=0.42, 95% CI 0.20 to 0.88 for the clerical/support social class). None of the work-related factors, or becoming a caregiver, predicted drinking above limits, either by itself or in combination (tables 2 and 3).

Additional adjustment for baseline long-term illness and depressive symptoms had virtually no effect on the estimates presented in tables 2 and 3, for either of the two outcomes.

### Job factors, caregiving and smoking cessation

Being in the lowest social class was associated with lower likelihood of quitting smoking (OR=0.42; 95% CI 0.27 to 0.63 in comparison with the highest social class). There were no statistically significant associations between the likelihood of quitting smoking and becoming a caregiver, nor between the likelihood of quitting smoking and job factors (table 2). At the same time, the effect of becoming a caregiver on quitting smoking was also dependent on work factors (table 3). Compared with those who did not become a caregiver and had average levels of social support, caregivers reporting high levels of social support were more likely to quit smoking (OR=2.99; 95% CI 1.01 to 8.86). Caregivers with low job demands were also more likely to quit smoking than non-caregivers with low job demands (OR=2.92; 95% CI 1.07 to 7.92), but not compared with non-caregivers with average levels of job demands (OR=2.24; 95% CI 0.83 to 6.06).

The estimates presented in tables 2 and 3 remained virtually unchanged after additional adjustment for baseline long-term illness and depressive symptoms.

### Job factors, caregiving and exercise

Low decision latitude and low socioeconomic status were associated with higher likelihood of reducing exercise below the recommended amount (table 2). Changes in exercise were, however, not related to job demands or social support at work. Becoming a caregiver was also not related to changes in exercise, and this was the case regardless of psychosocial working conditions at baseline (table 3). Additional adjustment for baseline long-term illness and depressive symptoms had virtually no effect on the estimates.

## Discussion

This paper investigated the combined effect of becoming a caregiver to an aged or disabled relative and adverse psychosocial conditions at work on changes in alcohol consumption, smoking and exercise. Our findings show that the extent to which becoming a caregiver affects changes in these behaviours may depend on psychosocial factors at the caregivers' work place prior to the increased family demands.

Previous literature on informal caregiving has documented both positive and negative health effects.^14^ ^15^ Accordingly, in our study, becoming a caregiving was not associated with adverse changes in health behaviours when considered in isolation. However, a combination of becoming a caregiver with low decision latitude at work, or with low social class, made it more likely that participants would increase the frequency of alcohol consumption. Furthermore, those who became caregivers were more likely to quit smoking if job demands were low and social support at work was high. These findings suggest that work factors such as collegial social support may help caregivers cope with the increased burden associated with caregiving. At the same time, the unfavourable conditions such as high demands and low latitude may exacerbate the effects of caregiving burden.

Becoming a caregiver did not affect changes in exercise, either by itself or in combination with adverse psychosocial working conditions. Stressful events in private life or stressful conditions at work may affect health behaviour through reduced self-regulation and motivation. However, becoming a caregiver may also be linked to physical activity by a completely different mechanism. Recall that participants were not only asked about sports (eg, what one chooses to do, or not to do, in their free time), but also everyday household activity, which may be a necessity. Becoming a caregiver might leave people with less time and desire for leisure sports, but at the same time, providing care to an aged or disabled relative is likely to entail extra chores that involve physical activity (eg, cleaning and shopping).

Smoking cessation and increased physical activity are generally regarded as health protective factors. At the same time, relationship between alcohol consumption and health are more complex, as alcohol has been discussed as both a risk and a protective factor.^30^ While our findings regarding increased frequency of alcohol consumption may be interesting in terms of stress-coping mechanisms, they may have less clear implications for public health interventions. We also included a more clinically relevant measure of alcohol consumption, namely increasing drinking to levels above recommended maximum. This outcome was not related to any of our exposures except social class, reflecting a previously established association between higher social class and higher levels of alcohol consumption.^31^ However, this measure was based on the number of drinks in the past week, and it is possible that the differences between baseline and follow-up reports reflect random weekly fluctuations.

Because of the design of the Whitehall II study with follow-ups conducted 2–3 years apart, we do not have precise information on the timing of becoming a caregiver and changes in health behaviours. Some of the changes in behaviour might have happened right after the participants assumed caregiving roles, but as they adjusted to their new tasks, they might have returned to their initial behaviours. Such changes would not have been captured in our data. Furthermore, Whitehall II is based on a sample of civil service workers, and thus, we may not have captured the whole range of psychosocial occupational exposure and socioeconomic position. This may have led to underestimation of potential interactive effects of work and family factors on changes in health behaviours. Finally, phases 3 and 4 of the Whitehall II study do not have more detailed information about the nature of caregiving, nor about the consequences of becoming a caregiver for the workers' employment (eg, whether their workload changed as a consequence of becoming a caregiver). These factors likely influence the work-family interactions investigated in the present study and need to be considered in the future research. For example, if some of those who became caregivers stopped working, the working conditions prior to becoming a caregiver probably had less effect on later health behaviours than in those who continued to work despite the changes in family situation. This may have resulted in an underestimation of the effects of working conditions on health behaviours after becoming a caregiver in our study. Despite these limitations, the findings of the present study contribute to the emerging literature on work-life interactions in predicting health-related outcomes.

In conclusion, we found that work-related factors influenced whether or not becoming a caregiver resulted in changes in alcohol consumption and smoking. This underscores the idea that in order to understand the true effects of stress, studies need to simultaneously consider stressors from different life domains. It also points to the importance of a supportive and well-balanced work environment as a resource for people exposed to burdens outside work.

**What is already known on this subject?**

- Stress is known to adversely affect health behaviour. Providing care to an aged or disabled relative may be associated with considerable stress, however, whether or not becoming a caregiver leads to adverse changes in health behaviours is not certain. The present paper investigated whether effects of becoming a caregiver on changes in alcohol consumption, smoking and exercise depended on caregivers' working conditions.

**What this study adds?**

- The results of the study showed that those who became caregivers and had low control over work-related decisions, or belonged to a low social occupational class, were more likely to increase the frequency of alcohol consumption than those who did not become caregivers. We also found that caregivers were more likely to quit smoking if their job demands were low or if social support at their workplace was high. These results suggest that supportive and well-balanced work environment may be an important resource for people exposed to burdens outside work.
